# Clinical Severity, Pre- and Post-Explantation Cytokine Profile and SEM/EDX-Defined Corrosion-Related Findings in Advanced Peri-Implantitis: Preliminary Results from the PERI-EDU Prospective Exploratory Clinical Study

**DOI:** 10.3390/jfb17080395

**Published:** 2026-08-11

**Authors:** Marzena Dominiak, Alicja Baranowska, Jacek Matys, Artur Pitułaj, Barbara Sterczała, Eduard Valmaseda-Castellón, Ricardo Castro Alves, Piero Papi, Umberto Romeo, Wojciech Simka, Artur Maciej, Julia Kensy, Paweł Kubasiewicz-Ross

**Affiliations:** 1 Department of Oral Surgery, Wroclaw Medical University, 50-425 Wroclaw, Poland; marzena.dominiak@umw.edu.pl (M.D.); alicja.baranowska@umw.edu.pl (A.B.); artur.pitulaj@umw.edu.pl (A.P.); barbara.sterczala@umw.edu.pl (B.S.); 2 Department of Odonto-Stomatology, Faculty of Medicine and Health Sciences, University of Barcelona, 08907 Barcelona, Spain; eduardvalmaseda@ub.edu; 3 Egas Moniz Center for Interdisciplinary Research (CiiEM), Egas Moniz School of Health & Science, 2829-511 Almada, Portugal; ricardocastroalves@gmail.com; 4 Department of Oral and Maxillofacial Sciences, Sapienza University of Rome, 00161 Rome, Italy; piero.papi@uniroma1.it (P.P.); umberto.romeo@uniroma1.it (U.R.); 5 Department of Inorganic Chemistry, Analytical Chemistry and Electrochemistry, Faculty of Chemistry, Silesian University of Technology, 44-100 Gliwice, Poland; wojciech.simka@polsl.pl (W.S.); artur.maciej@polsl.pl (A.M.); 6 Department of Pediatric Dentistry and Preclinical Dentistry, Wroclaw Medical University, 50-425 Wroclaw, Poland; julia.kensy@umw.edu.pl

**Keywords:** peri-implantitis, dental implants, explantation, IL-6, TNF-α, cytokines, SEM/EDX, corrosion, titanium particles

## Abstract

Background: Peri-implantitis is a chronic condition that reflects an interaction between local tissue destruction, systemic inflammation and corrosion-related implant alterations. This preliminary prospective exploratory clinical study aimed to characterize the systemic cytokine profile of patients with advanced peri-implantitis requiring explantation and to explore associations between clinical severity, cytokine behavior and scanning electron microscopy/energy-dispersive X-ray spectroscopy (SEM/EDX)-defined corrosion-related findings. Methods: Thirteen patients with active advanced peri-implantitis were included. Probing depth and cone-beam computed tomography (CBCT)-based bone loss were assessed at four implant surfaces. Peripheral blood was collected before and 4 weeks after explantation. Serum interleukin-1 beta (IL-1β), interleukin-6 (IL-6), interleukin-8 (IL-8), interleukin-10 (IL-10), interleukin-12p70 (IL-12p70), tumor necrosis factor alpha (TNF-α), high-sensitivity C-reactive protein (hsCRP), and fibrinogen were analyzed. Explanted implants underwent SEM/EDX assessment and were classified according to the presence or absence of SEM/EDX-defined corrosion-related findings. Results: IL-6 showed the strongest and most consistent associations with clinical and radiographic severity, whereas hsCRP and fibrinogen were not similarly related. Cytokines did not show a uniform post-explantation decrease after explantation. SEM/EDX-defined corrosion-related findings were not associated with higher baseline cytokines, but exploratory subgroup analysis suggested a divergent TNF-α trajectory. Conclusions: These findings suggest that IL-6 may reflect the clinical inflammatory burden of advanced peri-implantitis, while corrosion-related findings may be associated with post-explantation TNF-α behavior. Larger controlled studies are required.

## 1. Introduction

Peri-implantitis is a chronic inflammatory disease affecting the tissues around osseointegrated dental implants. It is characterized by an inflammation of the peri-implant mucosa leading to probing depth increase, bleeding or suppuration on probing and progressive loss of supporting bone [1,2,3]. In advanced cases, peri-implantitis may become refractory to treatment and may finally require implant explantation [3]. This makes peri-implantitis a clinically important complication, not only because it affects implant survival, but also because implant removal is often associated with further tissue destruction and the need for additional treatment [1,2,3].

The importance of this condition is increasing. Dental implant therapy is widely used worldwide as a predictable treatment option for partial and complete edentulism, and consequently, the number of patients treated with dental implants continues to rise [4]. This results in an increase in the total number of this biological complication. The prevalence of peri-implantitis has often been reported in the range of approximately 15–25%. Therefore, this condition should be considered not only an individual treatment failure, but also an emerging clinical and public health problem [5,6,7].

Although peri-implantitis is usually diagnosed and treated as a local disease, it may also be associated with systemic inflammatory changes. Associations between peri-implantitis and systemic inflammatory markers, in-cluding C-reactive protein (CRP), interleukin-6 (IL-6), and changes in blood count parameters, have been reported [8]. Interventional studies also suggest that peri-implantitis treatment may influence systemic inflammatory mediators. For example, non-surgical therapy has been associated with changes in peripher-al CRP, IL-6, and tumor necrosis factor alpha (TNF-α) levels [9]. However, most available studies evaluated patients at a single time point or focused on non-surgical treatment. Patients requiring implant removal represent a more severe clinical phenotype, and the short-term systemic cytokine response before and after explantation remains poorly described.

Another important issue is corrosion-related alteration of the implant surface. Titanium and titanium alloys are widely used because of their favorable mechanical properties, biocompatibility and passive oxide layer protecting inner layers from corrosion. However, the oral environment is complex. Biofilm, inflammation, low pH, fluoride-containing products, micromovements, mechanical wear, crevice conditions, and tribocorrosion may disturb the protective titanium oxide film and promote corrosion-related changes [10,11,12,13,14]. In peri-implantitis, titanium ions and particles have been described in peri-implant tissues [11,12]. Such findings suggest that corrosion is not only a material-related phenomenon but may also become biologically relevant in the diseased peri-implant environment, as these products can interact with resident and infiltrating immune cells. Macrophages are especially important because they are able to phagocytose particles, thereby releasing pro-inflammatory mediators, including TNF-α, IL-1β, and IL-6 [14,15,16]. This mechanism may link implant corrosion-related findings with a sustained local and systemic immune-inflammatory phenotype. However, this relationship is complex. Corrosion-related findings may not directly mirror the baseline cytokine profile, because systemic inflammation is also influenced by clinical disease severity, host response, metabolic status and the timing of sampling [14,15,16].

Studies of explanted implants have mainly focused on microbiology, surface morphology, or material degradation, whereas studies of systemic inflammatory markers have generally evaluated non-surgical peri-implantitis treatment [8,9,11,17]. Data combining paired serum cytokine measurements before and after implant explantation with SEM/EDX assessment of corrosion-related findings from the same patients remain limited.

The primary aim of this preliminary prospective study was to evaluate whether the clinical and radiographic severity of advanced peri-implantitis was associated with baseline serum cytokine levels. The secondary aim was to explore whether SEM/EDX-defined corrosion-related findings were associated with baseline serum cytokine levels or with their changes 4 weeks after explantation. We hypothesized that greater clinical and radiographic disease severity would be associated with higher baseline serum cytokine levels and that implant explantation would be fol-lowed by measurable changes in serum cytokine levels at the 4-week follow-up.

## 2. Materials and Methods

### 2.1. Study Design and Setting

This was a prospective, nonrandomized, single-group exploratory clinical study performed as part of the scientific section of the PERI-EDU project. The study is reported in accordance with the Transparent Reporting of Evaluations with Nonrandomized Designs (TREND) statement, and the completed TREND checklist is provided as non-publish material [18]. The project was conducted within the Erasmus+ KA220-HED consortium.

Patients were recruited and treated at the Department of Oral Surgery, Wroclaw Medical University, Wroclaw, Poland. The study included patients with active, advanced peri-implantitis who were considered for implant explantation.

The study was conducted in accordance with the Declaration of Helsinki. The protocol was approved by the Bioethics Committee at Wroclaw Medical University, Poland (KB 601/2024). The study was registered at ClinicalTrials.gov under the identifier NCT07012915. All participants provided two forms of written informed consent: one for the explantation procedure and one for participation in the study. The study was conducted under the General Data Protection Regulation (GDPR).

**Figure 1:**
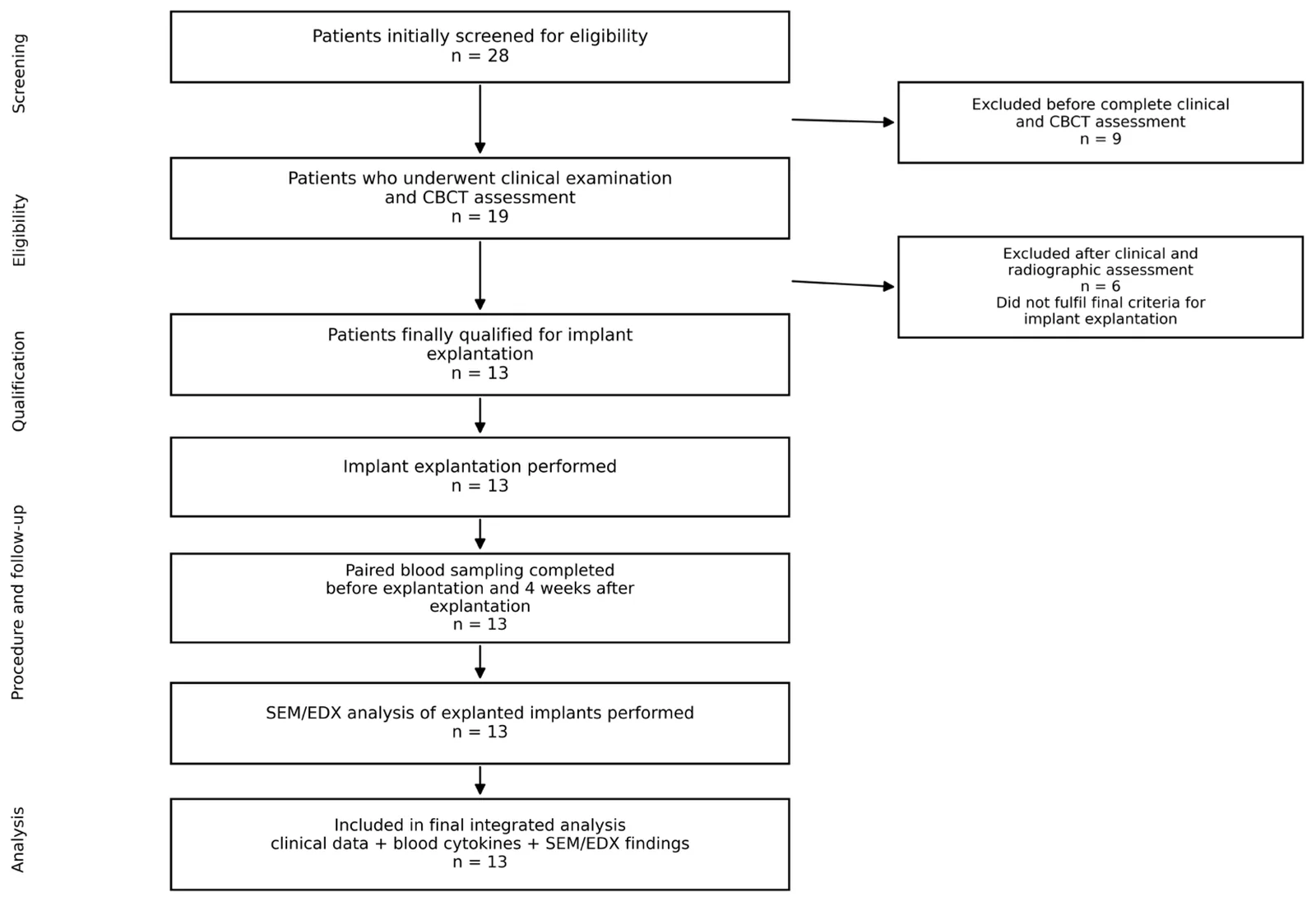
Flow diagram of patient enrollment.

Participant flow is presented in a structured diagram to transparently report patient screening, eligibility assessment, explantation, follow-up, and inclusion in the final analysis (Figure 1). Twenty-eight patients were initially screened for eligibility in the prospective PERI-EDU cohort. Nineteen patients underwent clinical examination and CBCT assessment. After clinical and radiographic assessment, 13 patients fulfilled final eligibility criteria for implant explantation and were included in the final integrated analysis (Figure 1).

### 2.2. Eligibility Criteria

Adult patients of both sexes were screened for participation. Patients were eligible if they had undergone previous dental implant treatment and presented with one implant affected by active, advanced peri-implantitis requiring implant explantation. If more than one implant was present, all remaining implants had to be clinically healthy, defined as probing depth (PD) ≤3 mm and negative bleeding on probing.

Active peri-implantitis was defined as PD greater than 6 mm at the affected implant site, together with positive bleeding on probing. Advanced peri-implantitis was defined as peri-implant bone loss radiographically exceeding 66% of implant length. Implant removal was considered clinically indicated if both criteria were met.

The exclusion criteria were age <18 years, pregnancy, general contraindications to oral surgical treatment, local contraindications to implant explantation, severe systemic disease increasing surgical risk or representing a contraindication to oral surgery procedures, autoimmune disease, and current or previous treatment with bisphosphonates or other antiresorptive medication.

Patients who did not meet the criteria for implant removal were referred for non-surgical or conservative surgical treatment and were not included in the present analysis.

### 2.3. Clinical Examination

All patients underwent clinical examination of the affected implant site. Probing depth was measured at four peri-implant surfaces: buccal, lingual or palatal, mesial, and distal. Bleeding on probing was recorded. Implant mobility was assessed clinically when present.

For statistical analysis, the following clinical variables were calculated: probing depth mean, defined as the mean probing depth from four surfaces; PD max, defined as the highest probing depth value around the implant; and PD ≥8 mm count, defined as the number of implant surfaces with a probing depth of at least 8 mm. These variables were used as indicators of local clinical severity.

### 2.4. Radiographic Assessment

Following clinical examination, all patients underwent cone-beam computed tomography (CBCT). Imaging was performed using the Planmeca Viso G7 system (Planmeca, Helsinki, Finland). A field of view (FOV) centered on the maxilla was used. The FOV measured 300 mm in width and 200 mm in height. Scans were analyzed using Romexis software version 6.5.2 (Planmeca, Helsinki, Finland).

**Figure 2:**
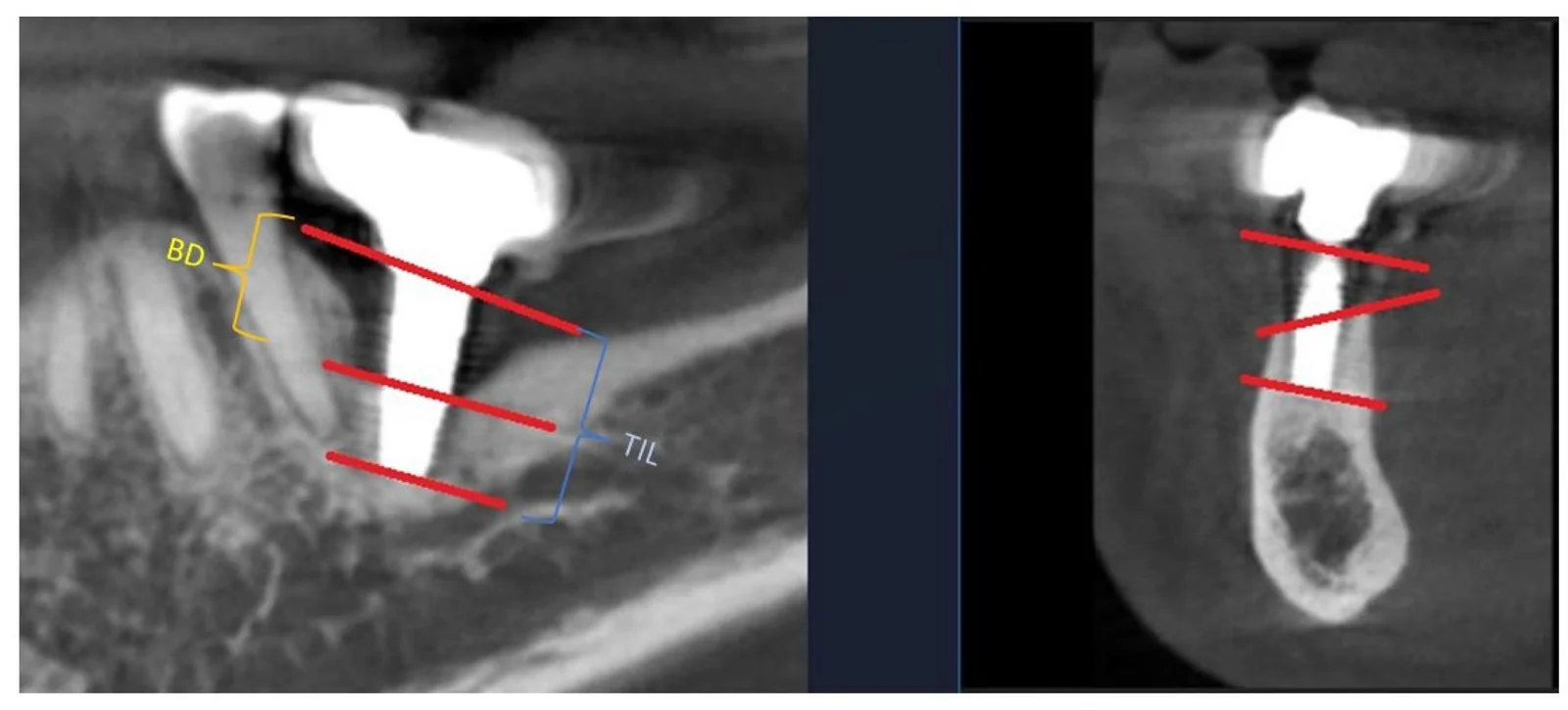
CBCT-based assessment of peri-implant bone loss.

Peri-implant bone loss was measured at four implant surfaces: buccal, lingual or palatal, mesial, and distal. Bone loss was expressed as a percentage of implant length. It was calculated as the ratio between the vertical distance from the implant platform to the bottom of the bone defect (BD) and the total implant length (TIL) (Figure 2).

For statistical analysis, the following radiographic variables were calculated: BL mean, defined as the mean bone loss percentage at four surfaces; BL max, defined as the highest bone loss percentage around the implant; and BL ≥80 count, defined as the number of implant surfaces with bone loss of at least 80%.

### 2.5. Blood Sampling and Laboratory Analysis

Peripheral venous blood was collected before implant explantation. A second blood sample was collected 4 weeks after explantation. Blood samples were collected from the antecubital vein and sent to a dedicated commercial analytical laboratory, Diagnostyka S.A. (Krakow, Poland). Blood collection, transport, processing, and storage were performed according to the standard operating procedures of the laboratory. The analysis focused on systemic immune-inflammatory and selected biochemical parameters. The primary laboratory variables were serum cytokine levels: interleukin-1 beta (IL-1β), interleukin-6 (IL-6), interleukin-8 (IL-8), interleukin-10 (IL-10), interleukin-12p70 (IL-12p70), and tumor necrosis factor alpha (TNF-α). Additional inflamma-tory and biochemical variables included high-sensitivity C-reactive protein (hsCRP) and fibrinogen.

### 2.6. Implant Explantation Procedure

All explantations were performed as standard oral surgical procedures. The prosthetic restoration was removed before surgery. After oral disinfection, local anesthesia was administered using 4% articaine with epinephrine 1:200,000 (Septanest 1:200,000; Septodont, Saint-Maur-des-Fossés, Paris, France). An envelope-type full-thickness mucoperiosteal flap was elevated to expose the peri-implant defect.

**Figure 3:**
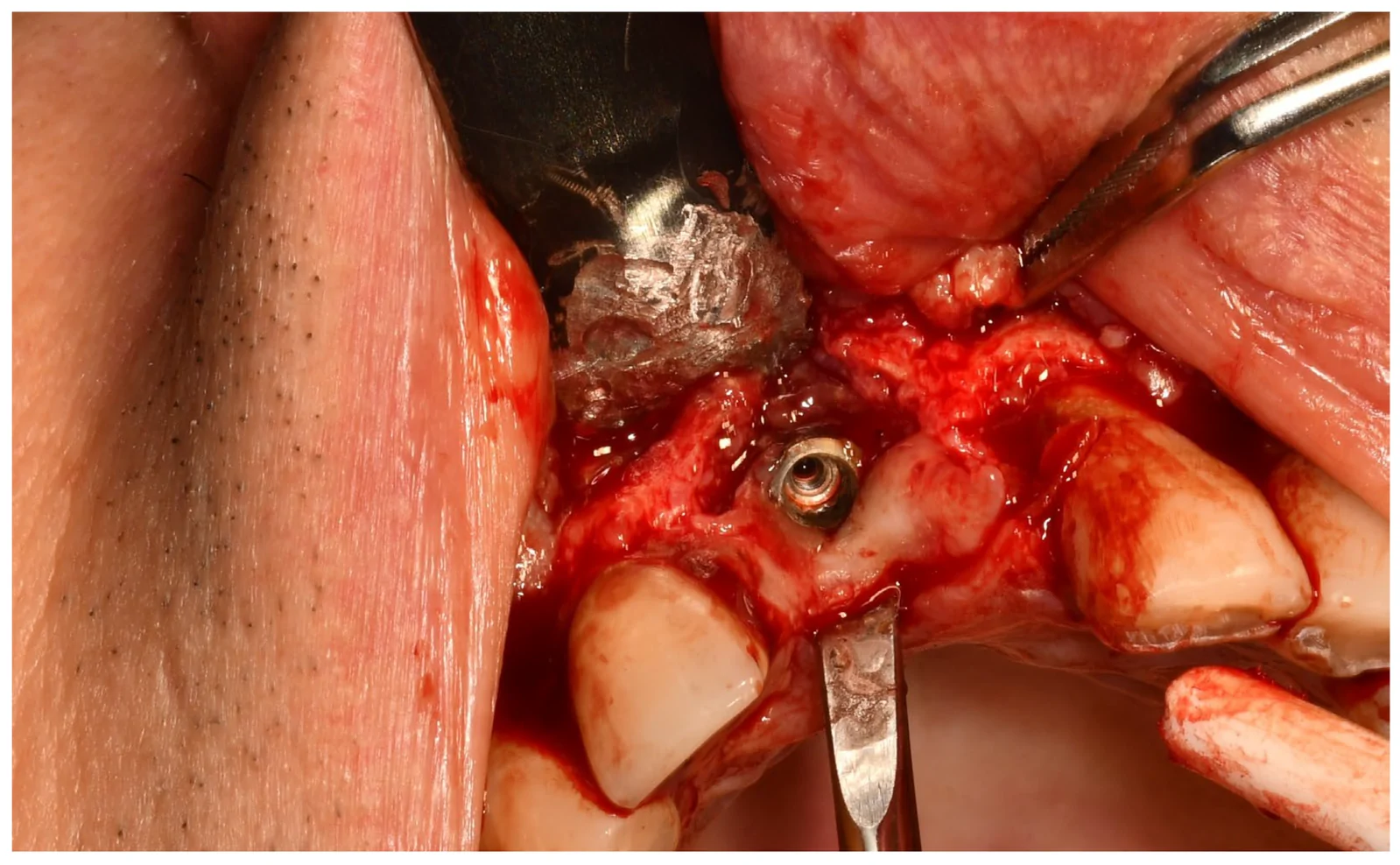
Intraoperative view after flap elevation around the failed implant.

Granulation tissue was initially removed with a curette. The peri-implant defect was debrided and irrigated with sterile saline (Figure 3).

**Figure 4:**
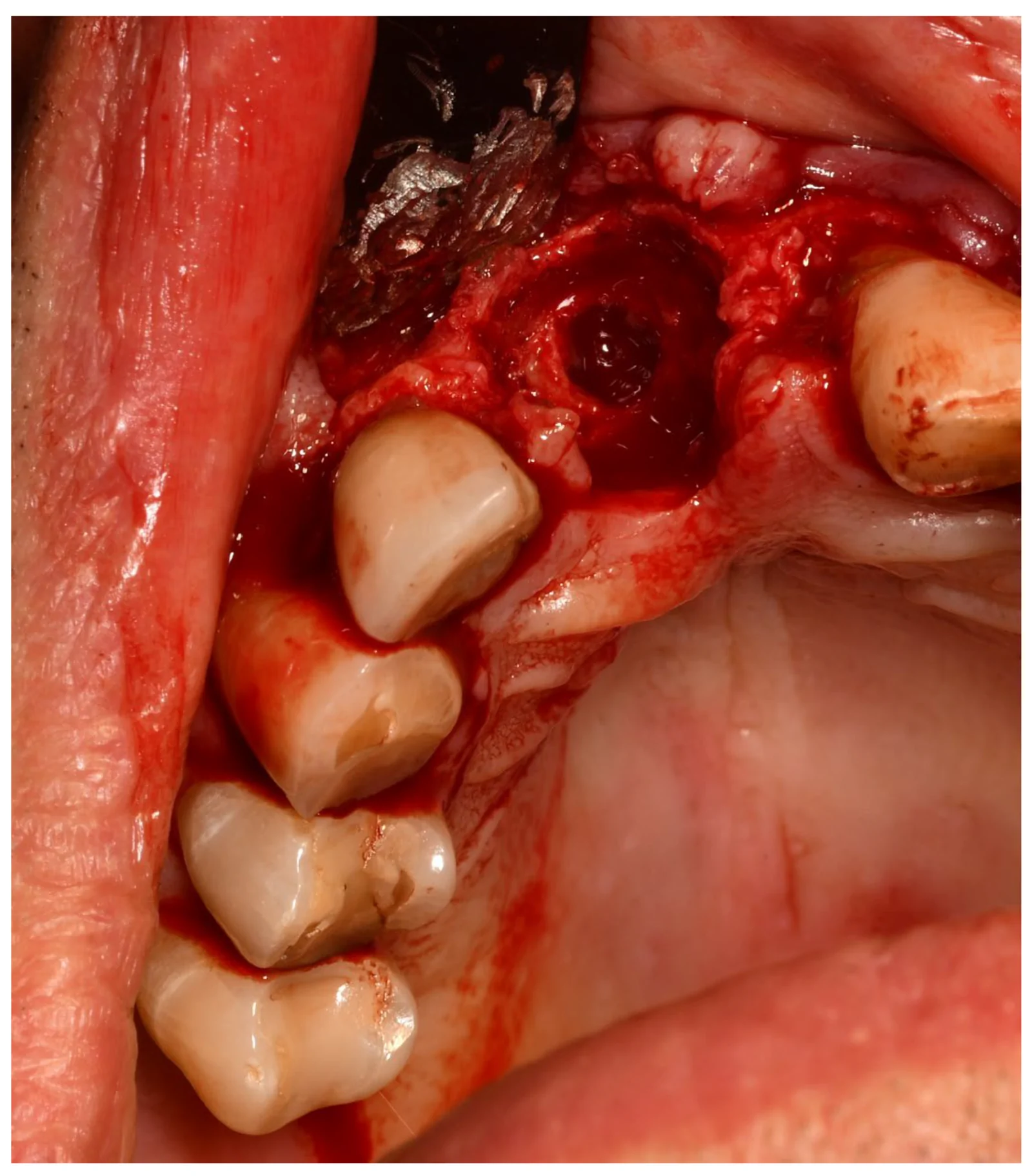
Post-explantation view of the peri-implant defect after implant removal and curettage.

The implant was then removed using reverse torque. Following implant removal, the defect was curetted again and irrigated (Figure 4). The wound was closed with single monofilament sutures. 

A control visit was performed one week after surgery for wound inspection and suture removal. A further follow-up visit was carried out 4 weeks after explantation. At this visit, peripheral blood was collected again for secondary analysis with the same variables analyzed previously.

### 2.7. SEM/EDX Analysis of Explanted Implants

After explantation, implants were transferred to the Department of Inorganic Chemistry, Analytical Chemistry and Electrochemistry, Faculty of Chemistry, Silesian University of Technology, Gliwice, Poland, for SEM/EDX analysis and corrosion-related assessment. SEM/EDX assessment was conducted by two calibrated examiners (W.S. and A.M.) who were blinded to the clinical and laboratory results.

Surface morphology was assessed using scanning electron microscopy, whereas EDX was used as qualitative supportive data for elemental characterization of the implant surface. In particular, the detection of fluorine and other elements supported the interpretation of selected SEM findings, including fluorine-related degradation, residual bone-related deposits, and possible abrasive particle residues.

SEM/EDX analysis was performed using a Phenom ProX scanning electron microscope equipped with an integrated energy-dispersive X-ray spectroscopy system (Thermo Fisher Scientific, Waltham, MA, USA). The microscope was equipped with a CeB6 electron source, a backscattered electron detector, and an integrated silicon drift detector (SDD) for energy-dispersive X-ray spectrosco-pyan integrated SDD-type EDS detector. Imaging and elemental analysis were performed using accelerating voltages of 5, 10, or 15 kV, depending on the analyzed area and required resolution.

Each implant was examined in five randomly selected areas. Images were obtained at different magnifications. The analysis focused on morphology, corrosion-related changes, coating delamination, coating cracking or chipping, fluorine-related degradation, tribocorrosion, crevice corrosion, and elemental composition.

### 2.8. Definition of SEM/EDX-Defined Corrosion-Related Findings

For the integrated clinical-laboratory-SEM/EDX analysis, findings were classified into two categories.

**Figure 5:**
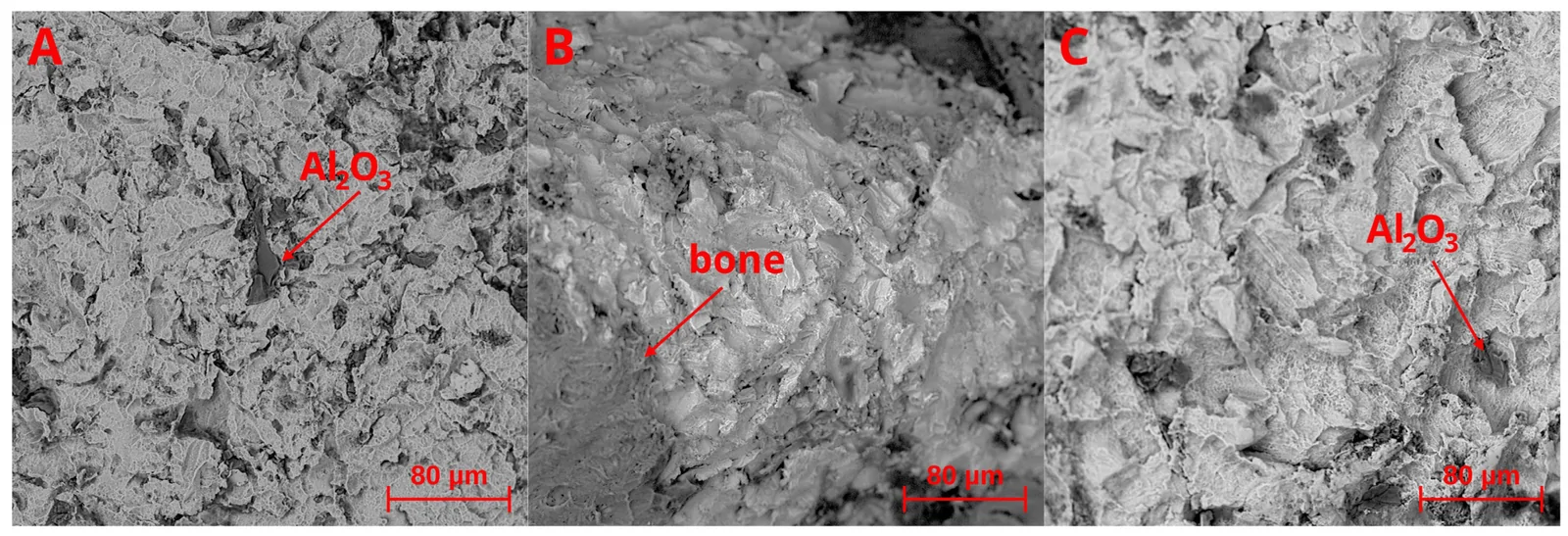
Representative SEM micrographs of implants without evident corrosion-related findings. On the implant surface, the corundum (**A**,**C**) and bone are visible (**B**). SEM images were acquired using a Phenom ProX SEM/EDX system at an accelerating voltage of 15 kV and a magnification of 1000×.

No evident corrosion-related findings were defined as the absence of reported corrosion, fluorine-related degradation, coating delamination, coating cracking, tribocorrosion or crevice corrosion (Figure 5).

**Figure 6:**
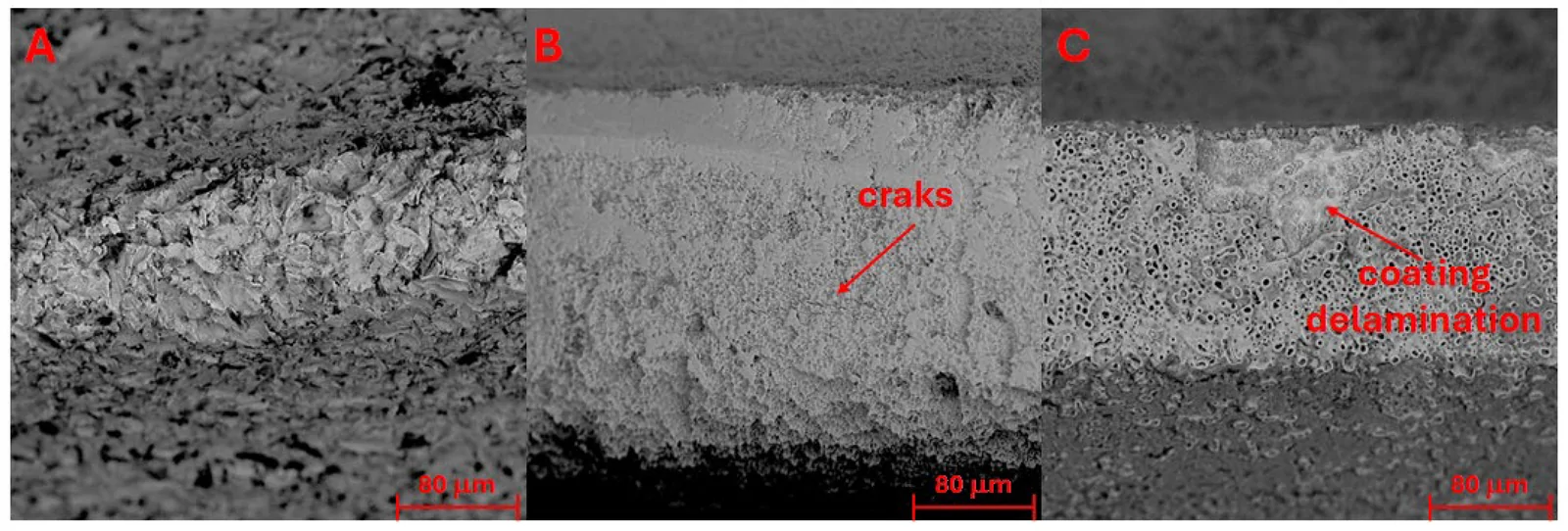
Representative SEM images illustrating corrosion-related findings on explanted implants: (**A**) corrosion-related etching/degradation; (**B**) tribocorrosion with crevice corrosion features; (**C**) coating delamination. SEM images were acquired using a Phenom ProX SEM/EDX system at an accelerating voltage of 15 kV and a magnification of 1000×.

The corrosion-related phenotype was defined as the presence of at least one of the following findings: fluorine-related degradation, possible hydrofluoric acid (HF)-related etching, coating delamination, coating cracking or chipping, tribocorrosion, or crevicecorrosion (Figure 6).

This broader category was used because these findings represent corrosion-related or corrosion-facilitating structural and chemical alterations of the implant. Such alterations may be associated with release of particles, ions, or coating fragments. These products may interact with macrophages and other immune cells and may contribute to cytokine-mediated immune activation.

### 2.9. Statistical Analysis

The final sample size was based on the number of patients with complete clinical, CBCT, paired laboratory, and SEM/EDX data. The cohort was considered sufficient for exploratory and for generating effect-size estimates for future controlled studies, but not for definitive subgroup comparisons. 

Because of the small sample size, statistical analysis was performed using non-parametric methods. Continuous variables were presented as medians and Q1–Q3 values. Categorical variables were presented as counts and percentages.

Spearman’s rank correlation coefficient was used to evaluate associations between clinical severity variables and laboratory markers.

The Wilcoxon signed-rank test was used for paired comparisons between pre- and post-explantation laboratory results. The Mann–Whitney U test was used to compare patients with and without SEM/EDX-defined corrosion-related findings. Fisher’s exact test was used for categorical variables when needed.

Because multiple exploratory comparisons were performed, Benjamini–Hochberg correction was applied to selected correlation analyses. Adjusted *p*-values were reported as q-values. For corrected correlation analyses, q-values below 0.05 were considered statistically significant. Statistical analyses were performed using STATISTICA software, version 13.3 (TIBCO Software Inc., Palo Alto, CA, USA).

Because this was a preliminary study, results were interpreted with emphasis on effect size, consistency of trends, and biological plausibility, rather than on *p*-values or q-values alone.

## 3. Results

### 3.1. General Characteristics of the Study Group

The study group consisted of seven women and six men. The median age was 52.0 years, with an interquartile range of 42.0–71.0 years. The age range was 32–79 years.

Based on SEM/EDX analysis, corrosion-related findings were present in five implants (38.5%) (Table 1).

### 3.2. Clinical and Radiographic Evaluation

All patients presented with advanced peri-implantitis. The median mean PD was 7.0 mm. The median maximum probing depth was 9.0 mm. The highest recorded probing depth was 10.0 mm.

Radiographic bone loss was also advanced. The median mean bone loss was 75.0% of implant length. The median maximum bone loss was 90.0% (Table 2).

### 3.3. Baseline Laboratory Profile Before Explantation

The baseline cytokine profile was heterogeneous. IL-6 showed marked interpatient variability and was later found to be the marker most closely associated with clinical severity. Classical inflammatory markers showed relatively low median values compared with the heterogeneity observed for cytokines (Table 3).

### 3.4. Association Between Clinical Severity and Baseline Cytokines

Spearman correlation analysis showed a strong relationship between peri-implantitis severity and circulating IL-6 before explantation.

PD mean correlated strongly with IL-6. Similar correlations were observed for PD max and the number of implant surfaces with PD ≥8 mm. Radiographic parameters showed the same pattern. BL mean, BL max, and the number of surfaces with BL ≥80% correlated with IL-6.

These correlations remained significant after Benjamini–Hochberg correction. IL-12p70 showed a weaker trend, mainly in relation to radiographic bone loss (Table 4). No consistent statistically significant associations were observed for the remaining cytokines, including IL-1β, IL-8, IL-10, and TNF-α, after correction for multiple testing. 

### 3.5. Threshold-Based Analysis of Disease Severity

Patients with higher clinical severity had higher IL-6 levels. When patients were divided according to PD mean ≥7.5 mm, the median IL-6 level was 6.42 pg/mL in the more severe group and 3.62 pg/mL in the less severe group. This difference was statistically significant.

A similar pattern was observed for BL mean ≥80%. Patients with BL mean ≥80% had a median IL-6 level of 6.71 pg/mL, compared with 3.78 pg/mL in patients with lower BL mean values.

IL-12p70 also differed according to BL mean ≥80%. This association was weaker than that observed for IL-6 but remained nominally statistically significant in the threshold-based analysis (Table 5). No consistent statistically significant associations were observed for IL-1β, IL-8, IL-10, TNF-α, hsCRP, or fibrinogen.

### 3.6. Pre- and Post-Explantation Laboratory Changes

Implant explantation was not followed by a uniform post-explantation decrease in systemic concentration of cytokines. IL-1β, IL-6, IL-8, IL-12p70, TNF-α, hsCRP, and fibrinogen did not show statistically significant paired changes.

IL-10 decreased in 9 of 13 patients, but this trend did not reach statistical significance. TNF-α decreased in 8 of 13 patients, but the paired change was also not statistically significant (Table 6).

### 3.7. Association Between SEM/EDX-Defined Corrosion-Related Findings and Baseline Inflammatory Markers

Patients with corrosion-related findings did not show greater clinical or radiographic severity than patients without evident corrosion-related findings. Median PD mean, PD max, BL mean, and BL max were not higher in the corrosion-related group. Baseline IL-6 and TNF-α were not higher in patients with corrosion-related findings. No important between-group differences were observed for IL-1β, IL-10, IL-12p70, hsCRP, or fibrinogen.

IL-8 was lower in the corrosion-related group than in the group without evident corrosion-related findings, and this comparison reached nominal statistical significance.

Overall, these findings indicate that SEM/EDX-defined corrosion-related findings were not associated with initial higher baseline clinical severity or a generally stronger systemic inflammatory profile (Table 7).

### 3.8. Corrosion-Related Findings and Post-Explantation Cytokine Trajectory

A different pattern was observed when cytokine changes after explantation were analyzed. Patients with SEM/EDX-defined corrosion-related findings showed a greater post-explantation change in TNF-α than patients without evident corrosion-related findings.

The median ΔTNF-α was +0.90 in the corrosion-related group and −1.16 in the group without evident corrosion-related findings. This difference was statistically significant. TNF-α increased in four of five patients with corrosion-related findings and decreased in seven of eight patients without evident corrosion-related findings.

A similar direction was observed for IL-6. The median ΔIL-6 was +1.64 in the corrosion-related group and −0.65 in the group without evident corrosion-related findings, but this difference was not statistically significant. The remaining cytokines and inflammatory markers did not show consistent changes between groups (Table 8).

## 4. Discussion

The present study suggests that advanced peri-implantitis requiring implant explantation may have a measurable systemic immune-inflammatory component. The most important observation was the close relationship between local disease severity and an increase in the systemic level of IL-6. This association was observed for both clinical and radiographic parameters of peri-implant severity assessment. At the same time, conventional inflammatory markers, such as hsCRP and fibrinogen, did not prove suitable for this particular approach.

This observation is consistent with previous studies showing that peri-implantitis may be associated with systemic inflammation. Yan et al. reported that patients with peri-implantitis may present higher systemic inflammatory markers, including CRP, IL-6, and white blood cell counts [8]. The strong association between IL-6 and peri-implantitis severity is biologically plausible. IL-6 is involved in the regulation of inflammatory signaling, acute-phase response, immune cell recruitment and bone resorption. In peri-implantitis, tissue destruction results from the interaction between biofilm, host immune response, osteoclast activation and inflammatory mediators. Therefore, IL-6 may reflect the inflammatory burden of active peri-implant tissue destruction more directly than downstream systemic markers. In the present analysis, IL-6 was related not only to probing depth but also to radiographic bone loss. This suggests that IL-6 may capture both soft-tissue inflammatory activity and the cumulative destructive component of the disease.

The weaker behavior of hsCRP and fibrinogen should also be interpreted carefully. These markers are general systemic indicators of inflammation and are influenced by many factors, including metabolic status, cardiovascular risk, obesity, smoking, infection, medication use and other chronic inflammatory conditions. In a small clinical cohort, these external influences may reduce their ability to reflect a localized inflammatory process around implants. IL-6 may therefore be more sensitive to active peri-implant tissue destruction than hsCRP or fibrinogen. This does not mean that classical markers are irrelevant. Rather, it suggests that cytokine-level assessment may provide more specific information in selected patients with advanced peri-implantitis.

Another important finding in our report was that cytokine levels did not consistently normalize 4 weeks after explantation. The 4-week follow-up was selected as an early post-explantation assessment point rather than as a time point of complete inflammatory resolution. A previous study of surgical peri-implantitis treatment showed significant changes in local protein biomarkers between 2 and 4 weeks [19]. Although that protocol as-sessed peri-implant crevicular fluid after reconstructive treatment rather than se-rum after explantation, it supports 4 weeks as a biologically active early healing interval. In theory, removing the affected implant together with the peri-implant granulation tissue should reduce the inflammatory burden. However, this pattern was not observed uniformly across the patients. Several explanations are possible. First, 4 weeks may be too short to observe stable systemic resolution after implant removal. The surgical wound is still undergoing healing and remodeling at this stage. Local tissue repair may itself influence cytokine production. Second, peri-implantitis may not be the only source of systemic cytokine activity. In the present protocol, patients were qualified only when one implant was affected by active advanced peri-implantitis, and any remaining implants had to be clinically healthy. Nevertheless, other oral or systemic inflammatory sources could not be fully excluded. Third, advanced peri-implantitis may be associated with a persistent immune-inflammatory phenotype that does not fully resolve within 4 weeks after removal of the local trigger.

Another possible explanation for the frequent cytokine abnormalities observed in the present cohort is the presence of a pre-existing systemic immune-inflammatory predisposition. Advanced peri-implantitis may not only induce systemic inflammation, but may also occur more frequently in patients with a host-related hyperinflammatory phenotype. Such patients may have higher baseline cytokine activity because of age, metabolic factors, smoking, obesity, periodontal history, cardiovascular risk, or other chronic inflammatory conditions. Therefore, elevated cytokine levels before and after explantation should not be interpreted as being caused exclusively by the peri-implant lesion. This interpretation is supported by studies showing that cytokine levels vary substantially even among apparently healthy individuals and are influenced by age, biological variability, lifestyle, and comorbidity burden [20,21,22,23]. In this context, persistent cytokine abnormalities after explantation may indicate that peri-implantitis is part of a broader systemic inflammatory background rather than an isolated local inflammatory process. 

This finding differs from studies assessing systemic changes after non-surgical peri-implantitis therapy. Liñares et al. showed that non-surgical treatment may reduce peripheral CRP, IL-6, and TNF-α after follow-up [9]. However, their study assessed conservative treatment, whereas our analysis focused on patients undergoing implant removal because of advanced disease. These are clinically different situations. In non-surgical treatment, improvement may reflect reduced biofilm burden and reduced mucosal inflammation over time. In explantation cases, the disease is more advanced, and the surgical procedure itself may create a short-term inflammatory response. Therefore, the absence of uniform cytokine reduction after 4 weeks should not be interpreted as treatment failure. It may instead indicate that systemic immune recovery after explantation is slower and more heterogeneous than local healing.

Serum cytokine levels provide information on systemic inflammatory activity but may not reflect inflammatory changes at an individual peri-implant site. Future studies should therefore combine serum measurements with peri-implant crevicular fluid (PICF) sampling from affected implants and from implants with healthy peri-implant tissues, either in the same patients or in a separate control group. Saxena et al. found significantly higher PICF IL-1β and IL-6 levels at peri-implantitis sites than at healthy implant sites and healthy teeth [24]. Zani et al. identified a distinct PICF multibiomarker profile, including TNF-α, that discrimi-nated diseased from healthy implant sites [25]. While, on contrary, in an intra-individual study, Jansson et al. found no significant differences in cytokine pro-files between peri-implantitis and periodontitis sites, supporting the inclusion of a periodontitis comparator when evaluating disease specificity [26]. 

The SEM/EDX evaluation adds another point to the interpretation. The corrosion-related findings were not associated with higher baseline cytokine levels. This is an important finding because it suggests that corrosion-related findings alone do not explain the systemic inflammatory profile in advanced peri-implantitis. Baseline IL-6 appeared to be driven mainly by clinical and radiographic severity rather than by the presence of corrosion-related changes on the implant surface.

In fact, the role of titanium degradation products in peri-implantitis remains debated. Titanium and titanium alloys are generally considered biocompatible, but their surface may change in the oral environment and become a trigger for the inflammatory process. Soler et al. reported that titanium corrosion may occur in clinical peri-implantitis conditions [11]. Safioti et al. found higher levels of dissolved titanium around implants affected by peri-implantitis [27]. Fretwurst et al. detected metal elements in peri-implantitis tissues [28]. Wilson et al. also described foreign bodies associated with peri-implantitis human biopsies, including titanium-containing material [29]. These studies support the concept that metal release and corrosion-related processes can occur in diseased peri-implant environments. More generally, laboratory studies of dental biomaterials have shown that surface roughness may influence bacterial adherence [30].

However, the presence of titanium particles or corrosion-related changes does not automatically prove that they initiated the disease. They may be a cause, a cofactor, or a consequence of peri-implantitis. Inflammation and biofilm may promote corrosion, while corrosion products may further stimulate inflammation. This bidirectional relationship is difficult to separate in clinical studies. Our results are consistent with this uncertainty. We did not find that corrosion-related findings were linked to higher baseline IL-6 or TNF-α. This argues against a simple model in which corrosion directly determines systemic cytokine levels at baseline.

The most intriguing finding related to corrosion was observed after explantation. Patients with corrosion-related findings showed a different TNF-α trajectory compared with patients without evident corrosion-related findings. TNF-α increased in most patients with corrosion-related findings, whereas it decreased in most patients without such findings. Although this observation must be interpreted as exploratory, it may have biological meaning.

TNF-α is closely associated with macrophage activation, inflammatory tissue destruction, and foreign-body-type immune responses. Titanium particles may interact with macrophages and modify their inflammatory phenotype. Kheder et al. showed that titanium particles may modulate macrophage and lymphocyte polarization in peri-implant gingival tissues [15]. Rakic et al. also investigated the immunopathological effect of titanium particles in peri-implantitis granulation tissue [31]. Reports by Asa’ad et al. and Chen et al. further support the concept that titanium particles and ions may contribute to peri-implant immune activation, although their exact role remains incompletely defined [12,13]. These studies provide a plausible biological background for the divergent TNF-α response observed in our cohort.

One possible explanation is that corrosion-related products remain in peri-implant tissues even after implant removal. Particles, ions, or coating fragments may be retained in granulation tissue, connective tissue, or bone defects. Related dental biomaterials research has also emphasized the importance of detailed surface characterization of modified coatings and of assessing the bio-logical, including cytotoxic and antimicrobial, properties of particulate-containing materials [32,33]. Surgical manipulation may also mobilize such material or increase contact between particles and immune cells. In this context, explantation may remove the implant itself but may not immediately eliminate the inflammatory effect of corrosion-related residues. This could explain why TNF-α increased in some patients with corrosion-related findings despite implant removal.

In contrast, in patients without evident corrosion-related findings, removal of the implant and inflamed peri-implant tissue may more directly reduce the inflammatory stimulus. This could explain the observed decrease in TNF-α in this group. This interpretation remains hypothetical, but it is consistent with the concept that corrosion-related products may act as modifiers of the immune response rather than as simple markers of disease severity.

The clinical meaning of these observations is still preliminary. Serum IL-6 may be a potential marker of advanced peri-implantitis severity, but it cannot be recommended for routine clinical use on the basis of this small cohort. Similarly, the association between corrosion-related findings and TNF-α trajectory should be treated as a hypothesis-generating finding. It suggests that corrosion-related implant changes may have immunological relevance, especially in relation to macrophage-driven cytokines, but this requires confirmation in larger studies.

This study has several limitations. First, the sample size was very small, and the subgroup analyses had limited statistical power. Therefore, the subgroup find-ings should be considered hypothesis-generating. Second, the study had no con-trol group. The absence of healthy individuals, patients with healthy or success-fully functioning implants, and patients with periodontitis limits the interpreta-tion and generalizability of the findings. Third, the follow-up was limited to 4 weeks and does not allow assessment of longer-term changes in serum cytokine levels. Fourth, no peri-implant tissue biopsies or PICF samples were collected. Therefore, serum cytokine levels could not be directly compared with local in-flammatory changes, and the proposed macrophage-related mechanism could not be confirmed histologically or immunohistochemically. Future controlled studies should include larger cohorts, longer follow-up, paired serum and PICF meas-urements, tissue-level immune profiling, and quantitative assessment of titanium particles or ions. 

Despite these limitations, the study provides a clinically relevant integrated perspective. Most previous studies evaluated peri-implantitis severity, systemic inflammation, titanium particles, or explanted implant surfaces separately [34]. In contrast, our study combined clinical parameters, paired peripheral blood cytokines, and SEM/EDX-defined corrosion-related findings in the same patients.

## 5. Conclusions

This preliminary prospective exploratory clinical study suggests that advanced peri-implantitis requiring explantation may be associated with systemic cytokine abnormalities. The strongest and most consistent systemic signal was the association between local clinical and radiographic severity and circulating IL-6.

Implant removal did not result in a uniform post-explantation cytokine decrease after 4 weeks, indicating that systemic inflammatory activity may persist beyond local surgical treatment or may reflect broader host-related inflammatory susceptibility. The SEM/EDX-defined corrosion-related findings were not associated with higher baseline cytokines, but it was linked to a divergent TNF-α trajectory after explantation. These findings require confirmation in larger controlled studies with longer follow-up and tissue-level immune profiling.

## Figures and Tables

**Table 1:** General characteristics of the final study group.

Variable	Value
Patients included in the final analysis	13
Corrosion-related findings present, n (%)	5 (38.5)
No evident corrosion-related findings, n (%)	8 (61.5)

SEM/EDX, scanning electron microscopy/energy-dispersive X-ray spectroscopy.

**Table 2:** Clinical and radiographic parameters of peri-implantitis severity.

Variable	Median	Q1–Q3	Range
PD mean [mm]	7.00	6.00–7.75	6.00–8.25
PD max [mm]	9.00	6.00–10.00	6.00–10.00
PD ≥8 surfaces [n]	1.00	0.00–2.00	0.00–2.00
BL mean [%]	75.00	70.00–82.50	70.00–85.00
BL max [%]	90.00	70.00–90.00	70.00–90.00
BL ≥80 surfaces [n]	1.00	0.00–3.00	0.00–3.00

PD, probing depth; BL, bone loss; Q1–Q3, first to third quartile.

**Table 3:** Baseline laboratory profile before implant explantation.

Variable	Median	Q1–Q3	Range
IL-1β [pg/mL]	2.43	1.92–2.82	1.49–9.86
IL-6 [pg/mL]	4.82	3.62–6.12	1.85–13.60
IL-8 [pg/mL]	5.11	4.66–6.00	2.73–16.30
IL-10 [pg/mL]	2.72	1.73–3.11	1.47–7.38
IL-12p70 [pg/mL]	1.97	1.88–2.54	1.11–9.85
TNF-α [pg/mL]	3.14	2.42–3.37	1.16–16.70
hsCRP [mg/L]	0.60	0.47–1.44	0.40–4.30
Fibrinogen [mg/dL]	252.00	201.00–309.00	182.00–365.00

**Table 4:** Main correlations between clinical severity and baseline systemic markers.

Marker	Clinical Variable	Spearman Rho	q-Value	*p*-Value
IL-6	PD mean	**0.859**	**0.00659**	**0.000168**
IL-6	PD ≥8 surfaces	**0.835**	**0.00659**	**0.000388**
IL-6	PD max	**0.831**	**0.00659**	**0.000428**
IL-6	BL ≥80 surfaces	**0.823**	**0.00659**	**0.000549**
IL-6	BL mean	**0.798**	**0.01039**	**0.001082**
IL-6	BL max	**0.742**	**0.02930**	**0.003662**
IL-12p70	BL mean	0.653	0.09585	0.01549
IL-12p70	BL ≥80 surfaces	0.651	0.09585	0.01597

q-values were calculated using Benjamini–Hochberg correction. Bold values indicate statistically significant associations after correction.

**Table 5:** Cytokine levels according to clinical severity thresholds.

Marker	Severity Threshold	Median, Severe	Median, Less Severe	*p*-Value
IL-6 [pg/mL]	PD mean ≥7.5 mm (n = 6 vs. n = 7)	**6.42**	**3.62**	**0.0012**
IL-12p70 [pg/mL]	PD mean ≥7.5 mm (n = 6 vs. n = 7)	2.67	1.90	0.0535
hsCRP [mg/L]	PD mean ≥7.5 mm (n = 6 vs. n = 7)	1.85	0.60	0.6131
Fibrinogen [mg/dL]	PD mean ≥7.5 mm (n = 6 vs. n = 7)	277.00	248.00	0.9452
IL-6 [pg/mL]	BL mean ≥80% (n = 5 vs. n = 8)	**6.71**	**3.78**	**0.0062**
IL-12p70 [pg/mL]	BL mean ≥80% (n = 5 vs. n = 8)	**2.79**	**1.89**	**0.0043**
TNF-α [pg/mL]	BL mean ≥80% (n = 5 vs. n = 8)	3.59	2.87	0.1709
hsCRP [mg/L]	BL mean ≥80% (n = 5 vs. n = 8)	0.60	0.66	0.9410
Fibrinogen [mg/dL]	BL mean ≥80% (n = 5 vs. n = 8)	252.00	272.50	0.5237

Bold values indicate nominally statistically significant differences.

**Table 6:** Changes in inflammatory marker serum levels following explantation.

Variable	Before Median	After Median	Median Δ	Direction	*p*-Value
IL-1β [pg/mL]	2.43	2.20	−0.06	6/7/0	0.6355
IL-6 [pg/mL]	4.82	4.38	+0.25	7/6/0	1.0000
IL-8 [pg/mL]	5.11	5.49	+0.14	7/6/0	0.7869
IL-10 [pg/mL]	2.72	2.00	−0.35	4/9/0	0.1099
IL-12p70 [pg/mL]	1.97	1.90	−0.07	5/7/1	0.5186
TNF-α [pg/mL]	3.14	2.71	−0.83	5/8/0	0.3396
hsCRP [mg/L]	0.60	0.71	+0.05	8/4/1	0.3379
Fibrinogen [mg/dL]	252.00	267.00	0.00	6/5/2	0.5049

Δ values were calculated as post-explantation minus pre-explantation values. Direction indicates the number of patients with increase, decrease, and no change, respectively.

**Table 7:** Baseline demographic, clinical, radiographic and systemic inflammatory profile according to SEM/EDX-defined corrosion-related findings.

Variable	Corrosion-Related Findings Present, n = 5 Median	No Evident Corrosion-Related Findings, n = 8 Median	*p*-Value
Age	45.00 years	58.50 years	0.5089
PD mean	6.25 mm	7.25 mm	0.8803
PD max	7.00 mm	9.50 mm	0.3992
BL mean	70.00%	75.00%	1.0000
BL max	70.00%	90.00%	0.4990
IL-1β	2.43 pg/mL	2.40 pg/mL	0.8329
IL-6	3.31 pg/mL	4.90 pg/mL	0.2222
IL-8	4.66 pg/mL	5.89 pg/mL	**0.0451**
IL-10	3.10 pg/mL	2.42 pg/mL	0.8329
IL-12p70	2.07 pg/mL	1.90 pg/mL	0.7140
TNF-α	2.73 pg/mL	3.29 pg/mL	0.2844
hsCRP	1.11 mg/L	0.60 mg/L	0.5054
Fibrinogen	297.00 mg/dL	250.00 mg/dL	0.9433

Comparisons were performed using the Mann–Whitney U test. *p*-values are nominal and should be interpreted descriptively because of the small sample size and exploratory design. Bold values indicate nominally statistically significant differences.

**Table 8:** Changes in inflammatory profile after explantation according to SEM/EDX-defined corrosion-related findings.

Variable	Median Δ, Corrosion-Related Findings Present, n = 5	Median Δ, No Evident Corrosion-Related Findings, n = 8	*p*-Value
TNF-α [pg/mL]	**+0.90**	**−1.16**	**0.0186**
IL-6 [pg/mL]	+1.64	−0.65	0.1709
IL-8 [pg/mL]	+1.94	−0.47	0.2844
IL-1β [pg/mL]	+0.34	−0.30	0.3543
IL-10 [pg/mL]	−0.20	−0.42	0.6216
IL-12p70 [pg/mL]	−0.07	−0.14	0.8329
hsCRP [mg/L]	+0.04	+0.15	0.2409
Fibrinogen [mg/dL]	0.00	+1.50	0.7691

Δ values were calculated as post-explantation minus pre-explantation values. Bold values indicate nominally statistically significant differences.

## Data Availability

The original contributions presented in the study are included in the article; further inquiries can be directed to the corresponding authors.

## Abbreviations

The following abbreviations are used in this manuscript:

BL	bone loss
CBCT	cone-beam computed tomography
CRP	C-reactive protein
EDS/EDX	energy-dispersive X-ray spectroscopy
GDPR	General Data Protection Regulation
hsCRP	high-sensitivity C-reactive protein
IL	interleukin
IQR	interquartile range
PD	probing depth
SEM	scanning electron microscopy
TNF-α	tumor necrosis factor-alpha
CONSORT	Consolidated Standards of Reporting Trials
FOV	field of view
HF	hydrofluoric acid
SDD	silicon drift detector
PICF	peri-implant crevicular fluid
